# Laparoscopic Common Bile Duct Exploration for Retrieval of Impacted Dormia Basket following Endoscopic Retrograde Cholangiopancreatography with Mechanical Failure: Case Report with Literature Review

**DOI:** 10.1155/2017/5878614

**Published:** 2017-07-13

**Authors:** J. W. O'Brien, R. Tyler, S. Shaukat, A. M. Harris

**Affiliations:** ^1^Department of Laparoscopic and Upper Gastro-Intestinal Surgery, Hinchingbrooke Healthcare NHS Trust, Hinchingbrooke Park, Huntingdon PE29 6NT, UK; ^2^Department of Gastroenterology, Hinchingbrooke Healthcare NHS Trust, Hinchingbrooke Park, Huntingdon PE29 6NT, UK

## Abstract

Dormia baskets are commonly used during endoscopic retrograde cholangiopancreatography (ERCP). One complication is basket retention, through impaction with a gallstone or wire fracture. We describe a case where the external handle of the basket snapped causing retained basket plus large gallstone impacted in the common bile duct (CBD). Following laparoscopic cholecystectomy, laparoscopic CBD exploration allowed direct stone fragmentation under vision with the choledochoscope. Fragments were removed using a choledochoscopic basket and Fogarty catheter, and the basket was withdrawn. Literature search identified 114 cases of retained baskets with management including shockwave lithotripsy (27%), papillary balloon dilatation (22%), open CBD exploration (11%), and one laparoscopic case.

## 1. Introduction

ERCP is used for relieving biliary obstruction caused by choledocholithiasis [1, 2]. Stone removal can be performed with a Dormia basket or balloon catheter, an approach that extracts up to 90% of CBD stones successfully [3, 4]. Dormia baskets most commonly consist of four stainless steel wires arranged at 90 degrees radially that are opened onto a stone to allow capture. When a stone is too big to be removed via the papillary orifice, some models permit mechanical lithotripsy, and rescue mechanical lithotripters are available for impaction. This has a success rate of 79% to 92% [5–8]. Overall complications of mechanical lithotripsy are between 6% and 13%, with basket impaction or wire fracture contributing up to 4% [5, 6, 9]. Retention of a Dormia basket in the biliary tree is a recognised complication [2]. This may be due to capture of a stone that is too large to permit removal, with subsequent impaction of the basket and stone. Alternatively, retention through loss of the ability to manipulate the basket can occur through fracture of the wires of the Dormia basket itself or fracture of a mechanical lithotripter, which can occur at an extra- or intracorporeal level. The impacted Dormia can cause cholangitis, pancreatitis, or migration, and no consensus exists on the optimal technique for removal [10]. We present laparoscopic management for a case of retained Dormia and review the available literature.

## 2. Case Report

**Figure 1:**
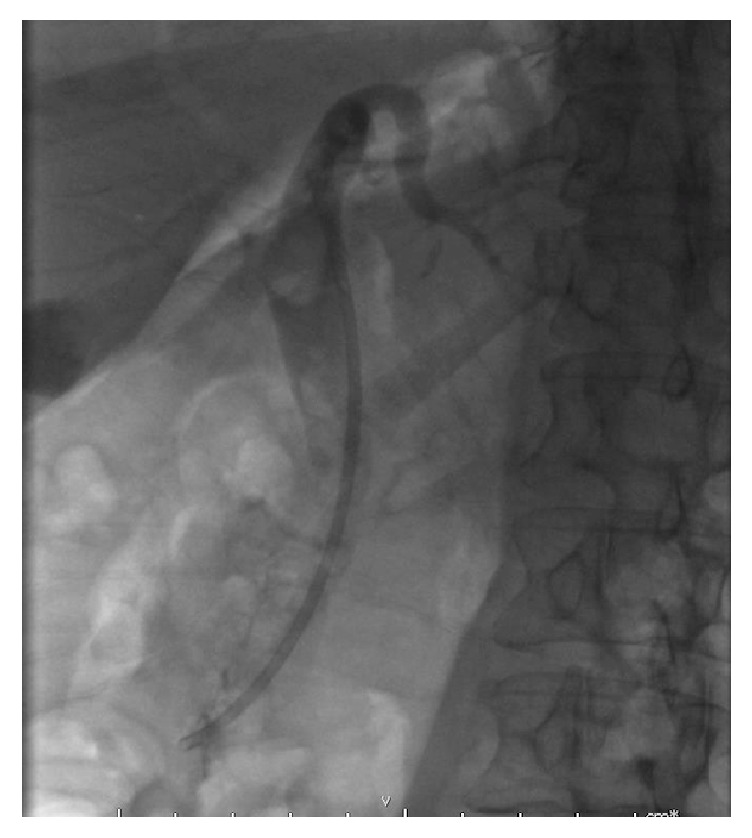
Gallstone seen near top of common bile duct.

**Figure 2:**
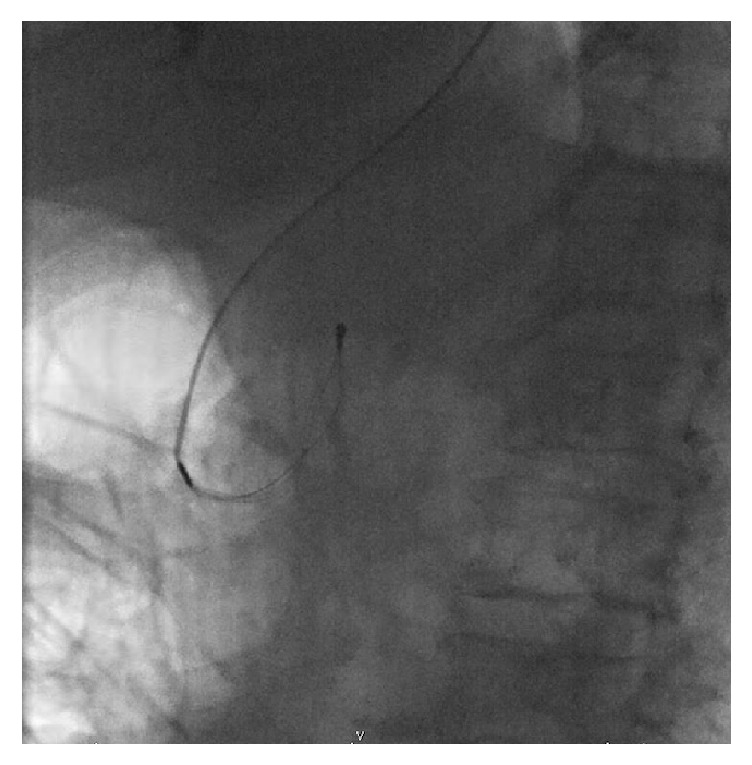
Dormia basket in CBD, after handle has broken (endoscope has been removed pending urgent surgery).

**Figure 3:**
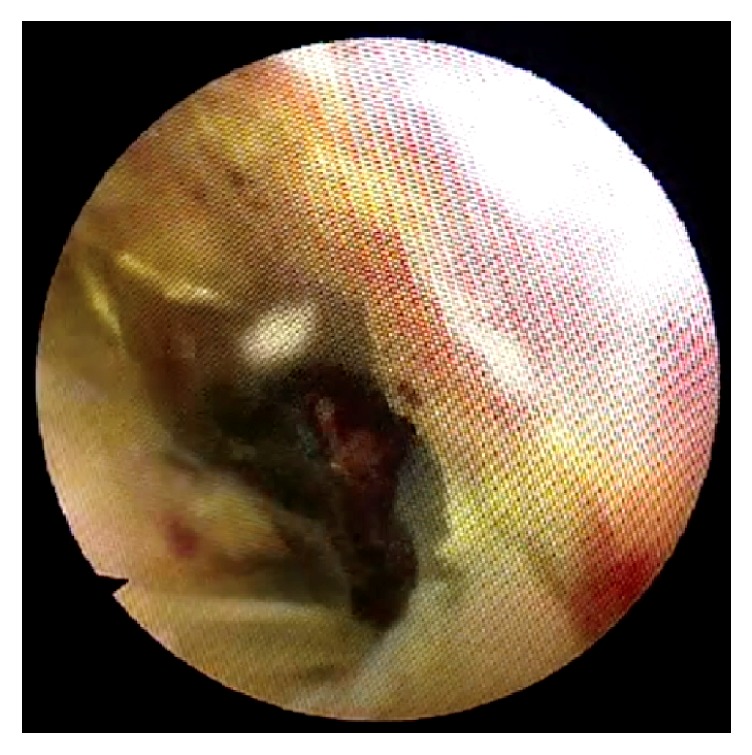
Choledochoscopic view of distal CBD. Large stone is seen within impacted ERCP Dormia basket.

**Figure 4:**
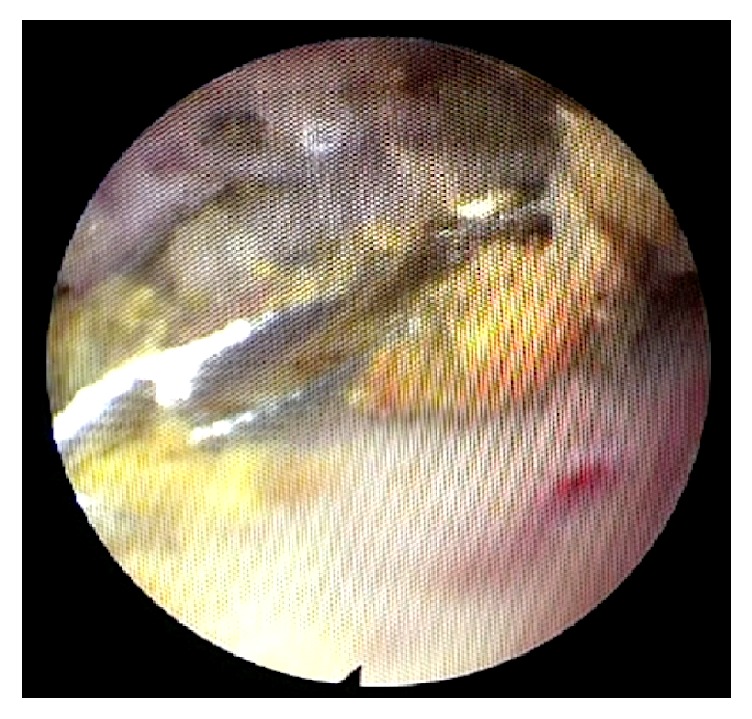
Debulked ERCP Dormia basket now collapsed and ready for removal, with few remaining stone fragments.

A 67-year-old Caucasian female with a body mass index of 30 was referred to clinic with symptoms of biliary colic. Past medical history included type 2 diabetes mellitus, hypertension, asthma, and previous total abdominal hysterectomy. Regular medications included bendroflumethiazide, metformin, omeprazole, ramipril, salbutamol, and simvastatin. She was a nonsmoker with minimal alcohol intake. Abdominal examination was normal and there were no signs of jaundice or anaemia. Ultrasound imaging revealed a common bile duct diameter of 13 mm, containing a 12 mm stone. Liver function tests showed bilirubin 13 (<21 mg/dl), alanine transaminase 26 (≤34 IU/L), and alkaline phosphatase 46 (20–140 IU/L). She underwent an ERCP with sphincterotomy, placement of a straight stent, and removal of several stones. One large stone that could not be removed with the Dormia basket was left in situ. During repeat ERCP three weeks later the large CBD stone (Figure 1) was engaged in a Dormia basket for mechanical lithotripsy but on cranking the lithotripter handle the wires snapped externally at the mouth (Figure 2). The patient was referred to upper gastrointestinal surgery and was taken as an emergency to the operating theatre the same day. A two-stage approach was taken following laparoscopic cholecystectomy. A 5 mm choledochoscope was introduced via a longitudinal choledochotomy and confirmed the presence of an impacted basket plus large stone at the ampulla (Figure 3). The choledochoscope itself was gently used to fragment the engaged stone into smaller pieces under direct vision. Next, a Fogarty balloon catheter and second Dormia basket* (Cook Medical, Ireland)* were used via the choledochoscope to remove most of the stone fragments laparoscopically. Having debulked the stone load (Figure 4), it was then possible to gently deliver the ERCP Dormia basket back into the duodenum without complication, which could then be removed orally. CBD clearance was confirmed and the choledochotomy closed with a 3-0 Vicryl continuous suture. After ERCP the patient was readmitted with acute pancreatitis and was hospitalised for 10 days, following which she made a full recovery.

## 3. Discussion

An extensive search of the English language literature in PubMed and MEDLINE® from 1950 onwards, including references, was carried out to obtain all published cases of retained biliary extraction baskets during ERCP for biliary or pancreatic stones. Search terms were “Dormia” or “basket” and “endoscopic retrograde cholangiopancreatography” or “ERCP”. 46 publications were identified, which included a total of 114 cases. One large case series by Thomas et al. (31 patients) [9] was not included in the following calculation because management of additional non-Dormia related complications was not described separately, leaving 83 cases. There were 42 (51%) cases of retention due to basket impaction on an impacted stone, without any wire fracture. There were 41 (49%) cases of basket retention due to wire fracture. 14 (34%) of the wire fractures were described subsequent to basket impaction. The wire fractures occurred extracorporeally, at the handle of a mechanical lithotripter in 13 (32%) and intracorporeally, along the guide wire or basket wires in 28 (68%), 25 of which were mechanical lithotripter wires. When specified, the CBD was the site of retention in 83% of cases.

The retained baskets were retrieved by a variety of strategies: extracorporeal shockwave lithotripsy (22), balloon dilatation (18), open surgery (9), a second Dormia basket (10), rescue mechanical lithotripter (5), exchange of metal wires (5), conservative management (2), exchange of metal sheaths (2), extension of sphincterotomy (2), laparoscopic surgery (2), laser lithotripsy (2), rat tooth forceps (2), goose neck snare (1), and papillotome to the stone (1). Each patient underwent an average of 1.4 procedures (including the original ERCP). In 33 papers (42 patients) definitive management of the common bile duct was described. 28 (67%) of these patients needed at least one further procedure to manage their choledocholithiasis following the ERCP, and 6 (14%) needed more than one further procedure. The average total number of extra procedures required to definitively manage choledocholithiasis per patient following ERCP was 0.67.

Recent case series report impaction of a Dormia basket during ERCP in as few as 0.6% and 0.8% of cases. Previously, it was reported in as many as 5.9% of cases [11–15]. Certain techniques increase the risk of basket retention. In one multicentre study of 31 patients, overall complications were three times higher following mechanical lithotripsy for pancreatic stones (11.6%) compared to biliary stones (3.6%), with fracture of the basket the most common complication [9]. Stone impaction, stone size, and a stone to bile duct diameter ratio greater than one are predictors of failed mechanical lithotripsy [5, 13]. Stone size over 20 mm is suggested as a contraindication for ERCP by some authors [16], but others suggest the ratio, which is increased by previous cholecystitis and cholangitis, is as important [5]. In such cases, García-Gallont et al. recommend using alternative methods as soon as the Dormia basket begins to deform in the stiffened common bile duct [17]. Typically basket impaction is encountered at the ampulla but case of impaction at the distal pancreatic duct in chronic pancreatitis is described [18] and less commonly the hepatic ducts [19] and a single case within the gallbladder itself [20].

The most commonly described management of an impacted Dormia basket is extracorporeal shock wave lithotripsy, perhaps reflecting the high proportion of cases reported from European centres. This results in high clearance after one session (92%), but subsequent endoscopy may be necessary to remove stone fragments and achieve definitive duct clearance [9, 12]. In the event of failure of laser lithotripsy, repeat laser shock wave lithotripsy is required with surgical exploration recommended if further lithotripsy fails [21]. Although the basket is visible under fluoroscopy, if cannulation of the bile duct is necessary for visualisation, this can be difficult if an impacted Dormia with stone is obstructing the distal bile duct. Importantly, such techniques are only available in certain centres, which can result in delays whilst the patient is transferred. Any delay in management can increase the severity of any subsequent pancreatitis, cholangitis and sepsis.

Rescue papillary balloon dilatation is an option if it has not already been carried out prior to basket impaction. The overall rate of bleeding following ERCP is 1.3% [22] and meta-analyses describe a similar 1.2% rate following combined sphincterotomy and balloon dilatation [23]. In a small case series six impacted baskets were successfully managed with balloon dilatation [15]. All six were done by the same tertiary centre endoscopist. Given the risk of perforation previously reported following balloon dilatation [24], this highlights the importance of specialist ERCP referral in such cases. Extreme care needs to be taken when using monopolar devices to extend the sphincterotomy in order to deliver an impacted basket, due to the risk of thermal injury caused by heat conduction through exposed basket wires [25].

Rescue mechanical lithotripsy was utilised successfully in five cases of retained Dormia. One centre describes four cases of impacted stone and Dormia successfully managed with “through the endoscope” rescue lithotripsy [26]. However, mechanical lithotripsy itself is associated with basket retention and wire fracture. 89% of intracorporeal wire fractures occurred along mechanical lithotripsy wires, and, similar to the case we report, there were 12 cases reporting lithotripsy handle fracture. Equipment failures occurred during use of “through the endoscope” and external or rescue mechanical lithotripters. There are not enough equipment details supplied in the literature to attempt subanalysis.

In 1993 Ng et al. described laparoscopic retrieval (via cholecystotomy) of a basket inadvertently impacted within the gallbladder itself but suggested that retention in the biliary tree was not amenable to laparoscopic rescue [20]. Laparoscopic common bile duct exploration, conducted in experienced centres, established itself in the late 1990s, showing similar success rates and morbidity to ERCP [27, 28]. Laparoscopic retrieval was first described in 2000 by Ainslie et al. [29]. This is only the second case to describe successful laparoscopic rescue. There was no mortality reported following open surgical management of a retained basket, which was the third most common method of retrieval in the literature.

## 4. Conclusion

This review describes the established risk of basket retention in the bile duct when using mechanical lithotripsy during therapeutic ERCP and further highlights a range of scenarios which may present to the endoscopist, including basket retention due to wire fracture. In centres with appropriate equipment, extracorporeal shockwave lithotripsy and papillary balloon dilatation for retained baskets are described in the literature, with minimal further procedures required to ensure treatment of choledocholithiasis. We describe a single operative treatment whereby, following laparoscopic cholecystectomy, laparoscopic CBD exploration via choledochotomy allowed retrieval of the retained basket and confirmation of duct clearance with choledochoscopy. We have shown the approach to be both feasible and safe and, where surgical expertise permits, can reduce the risk of further treatment sessions to manage residual common bile duct stones or debris. When encountering difficult (large or impacted) common bile duct calculi the endoscopist should consider which options are available at their institution in the event that a basket complication is encountered.
